# Use of Biotinylated Ubiquitin for Analysis of Rat Brain Mitochondrial Proteome and Interactome

**DOI:** 10.3390/ijms130911593

**Published:** 2012-09-14

**Authors:** Olga A. Buneeva, Marina V. Medvedeva, Arthur T. Kopylov, Victor G. Zgoda, Alexei E. Medvedev

**Affiliations:** 1 Orekhovich Institute of Biomedical Chemistry, Russian Academy of Medical Sciences, 10 Pogodinskaya street, Moscow 119121, Russia; E-Mails: olbun@yandex.ru (O.A.B.); a.t.kopylov@gmail.com (A.T.K.); vic@ibmh.msk.su (V.G.Z.); 2 Moscow State University, Moscow, 119991, Russia; E-Mail: marmed64@yandex.ru (M.V.M.)

**Keywords:** biotinylated ubiquitin, rat brain mitochondria, mitochondrial proteome, interactome

## Abstract

Applicability of *in vitro* biotinylated ubiquitin for evaluation of endogenous ubiquitin conjugation and analysis of ubiquitin-associated protein-protein interactions has been investigated. Incubation of rat brain mitochondria with biotinylated ubiquitin followed by affinity chromatography on avidin-agarose, intensive washing, tryptic digestion of proteins bound to the affinity sorbent and their mass spectrometry analysis resulted in reliable identification of 50 proteins belonging to mitochondrial and extramitochondrial compartments. Since all these proteins were bound to avidin-agarose only after preincubation of the mitochondrial fraction with biotinylated ubiquitin, they could therefore be referred to as specifically bound proteins. A search for specific ubiquitination signature masses revealed several extramitochondrial and intramitochondrial ubiquitinated proteins representing about 20% of total number of proteins bound to avidin-agarose. The interactome analysis suggests that the identified non-ubiquitinated proteins obviously form tight complexes either with ubiquitinated proteins or with their partners and/or mitochondrial membrane components. Results of the present study demonstrate that the use of biotinylated ubiquitin may be considered as the method of choice for *in vitro* evaluation of endogenous ubiquitin-conjugating machinery in particular subcellular organelles and changes in ubiquitin/organelle associated interactomes. This may be useful for evaluation of changes in interactomes induced by protein ubiquitination under norm and various brain pathologies.

## 1. Introduction

Ubiquitin is a 76-residue protein, which is widely distributed in all eukaryotic cells [1–5]. The carboxyl group of Gly76 of ubiquitin forms an isopeptide bond with, most typically, the ɛ-amino group of a lysine residue within substrates [6]. ATP-dependent ubiquitin modification of various protein targets determines the role of this protein in numerous intracellular processes including regulation of gene expression, cell cycle and division, stress response, elimination of damaged proteins, DNA repair, import of proteins to mitochondria, assembly of ribosomes, apoptosis, *etc.* [1–5].

The ubiquitination process includes several stages, which involve several enzymes: ubiquitin activating enzyme, ubiquitin-conjugating enzyme, and ubiquitin ligase [7–8]. Taking into consideration the regulatory role of protein-protein interactions controlling the ubiquitination process [1–9], interactome, it is clear that ubiquitin and other components of the ubiquitn conjugating machinery as well as ubiquitinated proteins do potentially interact with many protein partners and therefore form a particular ubiquitin.

Levels of ubiquitinated proteins and ubiquitin binding partners are usually rather low for detection and therefore there is a clear need for the use of various strategies for their enrichment [6]. Isolation and identification of ubiquitinated proteins usually employ affinity chromatography purification, proteolytic digestion of eluted proteins, and analysis by mass spectrometry. Trypsinolysis of ubiquitinated proteins yields a unique peptide from the ubiquitination site containing a lysine residue with an isopeptide-linked glycine-glycine sequence [9]. This signature peptide can be identified by mass analysis due to its mass shift of 114.1 Da and the lack of proteolytic cleavage of modified lysine residues.

Proteomic analysis of ubiquitinated proteins is frequently based on the incorporation of tagged ubiquitin, which is used for subsequent affinity purification of ubiquitinated proteins [10]. However, the tag-based approach requires genetic manipulation with cells and/or multicellular organisms and therefore is basically inapplicable for analysis of clinically relevant samples [10]. This problem is frequently solved by employment of ubiquitin antibodies [11–12] adapted for affinity purification with some success [10]. However, detection of ubiquitinated proteins by means of antibody-based detection methods usually provides information about ubiquitination of a particular protein and leaves out of consideration possible alterations in the ubiquitin interactome.

An alternative method effective for protein purification is biotin tagging. The biotin-avidin binding (*K*_D_ = 10^−15^ M) is the strongest known biochemical non-covalent interaction [13] and is resistant to much more stringent washes, minimizing nonspecific binding. Ubiquitin tagging by biotin usually involves genetic manipulations with microorganisms, cell cultures and whole macroorganisms [10]. Applicability of *in vitro* biotinylated ubiquitin for evaluation of endogenous ubiquitin conjugating activity and analysis of ubiquitin-associated protein-protein interactions in mammalian tissues and their subcellular fractions has not been investigated yet.

In this study we have performed proteomic profiling of proteins isolated using avidin-Agarose after incubation of rat brain mitochondrial fraction with biotinylated ubiquitin. Although it has been used in various studies on ubiquitination of soluble (non-mitochodnrial) proteins in different types of eukaryotic cells [10], this approach, however, has not been used either for analysis of ubiquitination of brain mitochondrial proteins or for analysis of the mitochondrial interactome. Previous studies have demonstrated that brain mitochondria do contain components of the protein ubiquitination machinery [14,15] and incorporation of exogenous ubiquitin into these organelles *in vitro* is accompanied by increased sensitivity of some enzymes to proteolysis [16,17].

## 2. Results

**Figure 1:**
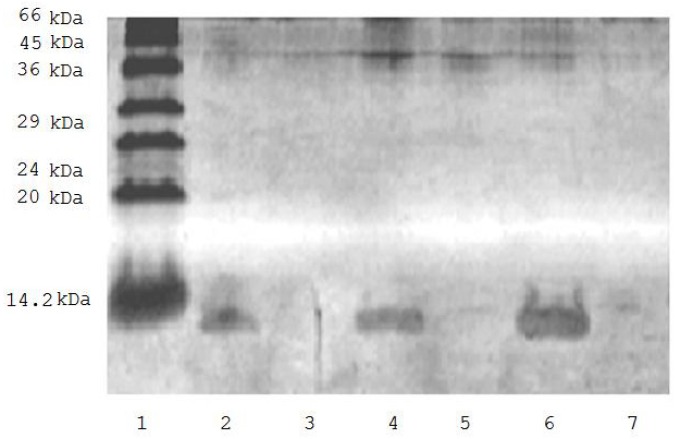
SDS-PAGE of solution of biotinylated ubiquitin before (tracks 2, 4, 6) and after incubation (tracks 3, 5, 7) with avidin-agarose. 1. low molecular weight markers; 2. 0.375 μg of biotinylated ubiquitin; 4. 0.75 μg biotinylated ubiquitin; 6. 0.150 μg of biotinylated ubiquitin; 3, 5, and 7 are the same as in 2, 4, and 6 but after incubation with avidin-agarose.

Figure 1 shows that incubation of biotinylated ubiquitin (0.9 mg/mL) with avidin-agarose suspension (1:1) resulted in complete elimination of the protein from the incubation medium (Figure 1) thus suggesting its effective binding to the sorbent. This allowed us to use the biotinylated ubiquitin for evaluation of the effect of ubiquitin on mitochondrial proteomic profiling.

Incubation of rat brain mitochondrial fraction with biotinylated ubiquitin followed by loading of cleared Triton X-100 lysates onto the avidin-agarose, intensive washing of the affinity sorbent and subsequent proteomic analysis of eluted proteins resulted in identification of 50 individual proteins (Table 1).

The same incubation of the rat brain mitochondrial fraction without biotinylated ubiquitin (control) followed by the same affinity chromatography fractionation resulted in identification of some highly abundant (and mostly) cytoskeletal proteins (Table 2).

All these proteins (Table 1) preferentially associated with both outer and inner mitochondrial compartments bound to avidin-agarose only after preincubation of brain mitochondria with biotinylated ubiqutin. They could be referred to the following functional groups of proteins/enzymes: (1) Proteins (enzymes) involved in carbohydrate metabolism and energy generation; (2) Proteins involved in cytoskeleton formation and exocytosis; (3) Antioxidant/protective proteins; (4) Proteins/enzymes involved in signal transduction and regulation of enzyme activity; (5) Proteins of fatty acid metabolism; (6) Transporters; (7) Ubiquitin and related proteins.

Search for specific ubiquitination signature masses (+114.04 Da) revealed several ubiquitinated proteins of both extramitochondrial and intramitochondrial compartments (Table 3). Some of them had more than one ubiquitination site. Thus results of these experiments suggest that *in vitro* ubiquitination by means of exogenous biotinylated ubiquitin involves both external and intrinsic mitochondrial proteins.

**Figure 2:**
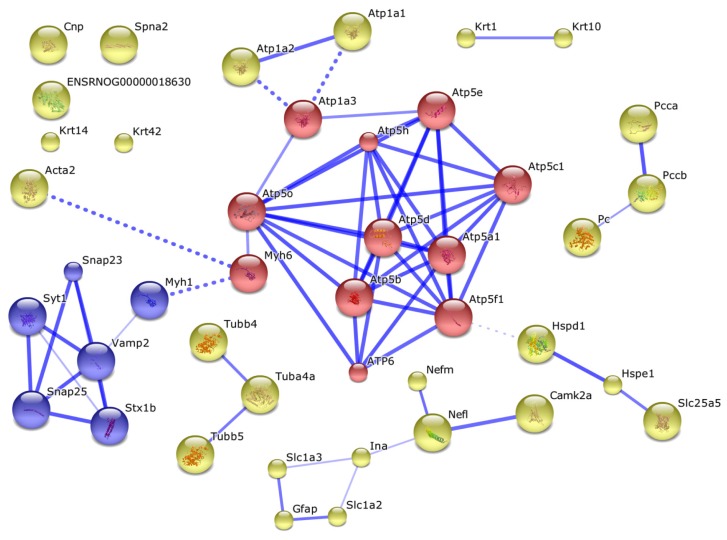
The interactome of identified proteins, containing ubiquitin signatures. Solid blue lines designate direct interactions between proteins, and dashed blue lines demonstrate the most confident interactions between the cluster of intramitochondrial proteins and clusters of extramitochondrial proteins involved in cytoskeleton formation and carbohydrate metabolism and other proteins. Acronym names of the proteins shown in this figure are listed in Table 3. Intramitochondrial proteins are shown as red circles. Proteins indirectly interacting with mitochondrial components via more than one linker protein are shown as unconnected circles.

Other proteins obviously formed complexes with the ubiquitination proteins or they could be involved into formation of mitochondrial interactome (Figure 2).

## 3. Discussion

Results of this study provide clear evidence that incubation of the rat brain mitochondrial fraction with biotinylated ubiquitin *in vitro* results in direct ubiquitination of some extramitochondrial and intramitochondrial proteins. In these experiments we have used the crude rat brain mitochondrial fraction, which has been originally used in pilot experiments performed to investigate the effect of exogenously added ubiquitin on proteolytic sensitivity of mitochondrial enzymes [16,17]. Since the employed system results in ubiquitination of both extramitochondrial (*n* = 10) and intramitochondrtial (*n* = 2) proteins it appears that under our experimental conditions components of the ubiquitin conjugation machinery associated with both extramitochondrial and mitochondrial compartments have used biotinylated ubiquitin. However, the proportion of directly ubiquitinated proteins represents not more than 20% of the total number of identified proteins specifically bound to avidin-agarose.

Previous proteomic studies of endogenous ubiquitination of human myocardial proteins demonstrated that more than 200 distinct proteins were bound to the affinity column (S5a-agarose) and only 27 proteins (*i.e.* less than 10%) were identified as directly ubiquitinated proteins [18]. Other proteins were obviously bound to the column in association with ubiquitinated proteins [18]. It should be noted that in accordance with data by Weekes *et al.* [18], we found alpha-chain of ATP synthase and myosin as direct targets for ubiquitination, while glyceraldehyde-3-phosphate dehydrogenase, creatine kinase M type which were found to be directly ubiquitinated in hearts of cardiac patients [18] lacked specific ubiquitin signature masses.

Using a transgenic mouse expressing octahistidine/Flag-tagged ubiquitin (HisF-Ub) in the heart Jeon *et al.* [19] identified 121 ubiquitinated proteins, including more than 40 mitochondrial proteins. Although about 10 proteins were also found in our study only two of them (alpha subunit of ATP synthase, sodium-potassium-transporting ATPase subunit alpha-3) contained specific ubiquitin signature masses.

Thus, it appears that *in vitro* incorporation of exogenous ubiquitin to rat brain mitochondria is accompanied by direct ubiquitination of some extramitochondrial and intramitochondrial proteins. However, it should be noted that among 12 identified proteins that contained specific ubiquitin signatures only pyruvate carboxylase and alpha subunit of ATP synthase are located in mitochondrial matrix or associated with the inner surface of the inner mitochondrial membrane, respectively. Although others have extramitochondrial localization, certain evidence exists that most of them (even plasma membrane proteins) may interact with mitochondria and thus participate in mitochondrial interactome formation (Figure 2). Our results suggest that ubiquitination of some proteins at both sides of the mitochondrial membranes significantly influences the mitochondrial interactome (evaluated in our experiments by the avidin-agarose affinity chromatography probing) (Figure 2). This is consistent with literature data on the dependence of mitochondrial functioning on functional competence of the ubiquitin-proteasomal machinery [20–22].

In order to minimize nonspecific adsorption of proteins onto the affinity sorbent we have employed the washing system (see Experimental Section 3.6), which is frequently used in affinity purification of antibodies [23]. Reliable detection of 50 proteins (Table 1) suggests that proteins bound nonspecifically to the affinity sorbent have been removed during the sorbent washing, and non-ubiquitinated proteins obviously form tight complexes either with ubiquitinated proteins or with their partners and/or mitochondrial membrane components. Results of the interactome analysis seem to support our viewpoint (Figure 2).

It should be noted that good evidence exists in the literature that some plasma membrane proteins interact with mitochondrial interactome. For example, vesicle-associated membrane protein 2 (Vamp 2), which is highly expressed in secretory vesicles and located in synaptic vesicle membrane, interacts with myosin heavy chain 4 associated with myosin heavy chain 6 [24,25]. Myosin 6 directly interacts with ATP-synthase subunit O. Considering this branch of the interactome (Figure 2) one can see that it can be further extended from Vamp 2 to synaptosomal-associated proteins 23 and 25 (SNAP23 and SNAP25) [26,27]; functional activity of this complex is regulated by synaptotagmin-1 associated with syntaxin [28]. Synaptotagmin-1, a protein associated with the vesicular membrane, is considered as a Ca^2+^ sensor for neurotransmitter release [29] and a regulator of Vamp 2 functioning. Synaptotagmin-1 also forms complexes with SNAP 23 and SNAP 25 proteins and thus promotes Vamp 2-dependent *O*-glycosylation [30]. These complex-forming proteins interact with of mitochondrial ATP synthase subunits via myosins, containing nucleotide-binding domains [31,32]. The interaction between actin and the ATP-synthase complex also involves myosin 6 subunits. Some syntaxin proteins may form a functional complex with the sodium-dependent glutamate/aspartate transporter 2 (GLT-1), which is strictly required for regulation of glutamante uptake by cAMP in the central nervous system [33]. This cAMP-dependent regulation of glutamante uptake is indirectly associated with mitochondrial ATP-synthase subunits and adenine nucleotide translocase (ANT) [34,35]. The glial fibrillary acidic protein (GFAP) plays a key role during the development of the central nervous system [36,37]; it is considered as a marker that distinguishes astrocytes from other glial cells [38,39]. GFAP acts as a link between Vamp 2 and SNAP 23/SNAP 25 proteins and also between ANT and synaptotagmin [40]. Impairments of such interactions have been recently demonstrated using a murine model of Alzheimer’s disease [41].

Thus, even brief consideration of the interactome data indicates that the non-ubiquitinated proteins co-isolated on avidin-agarose together with the biotin-tagged ubiquitinated proteins represent co-isolated components of mitochondrial interactome sub-complexes rather than nonspecific contaminants. Existence of such interactome sub-complexes is reasonably documented in the literature [24–41].

Thus, using biotinylated ubiquitin and proteomic methodologies supplemented by bioinformatic analysis, it is possible not only to find direct ubiquitination targets in rat brain mitochondria but also to get valuable information on the mitochondrial interactome influenced by protein ubiquitination.

Although biotinylated ubiquitin has limited (if any) applicability for *in vivo* studies, the proposed algorithm employing identification of both ubiquitinated proteins and specific protein complexes co-isolated with ubiquitinated proteins would be useful for mapping of certain mitochondrial (sub) interactomes.

## 4. Experimental Section

### 4.1. Chemicals

Avidin agarose, biotin, ubiquitin, DTT, iodoacetamide, trypsin, Tris (hydroxymethyl) aminomethane, guanidine hydrochloride, ammonium hydrocarbonate, potassium phosphate, sodium phosphate, sodium chloride, magnesium chloride, potassium chloride, Triton X-100, sucrose, EDTA, ATP, creatine phosphokinase, creatine phosphate, bacitracin, aprotinin, ACN, formic acid, CBB R-250, CBB G-250 were purchased from Sigma-Aldrich (USA).

Acrylamide, *N*,*N*′-methylenebisacrylamide, Low molecular weight protein standards, SDS, ammonium persulfate, TEMED were from Bio-Rad (USA).

### 4.2. Animals and Preparation of Brain Mitochondrial Fraction

Male Wistar rats (250 g–300 g) obtained from the Stolbovaya nursery (Russian Academy of Medical Sciences) were used in the experiments that were performed at least one week after their arrival from the nursery. Animals received a standard laboratory chow and water *ad libitum* and their decapitation was performed between 11.00 and 13.00. The brains were immediately dissected and homogenized in the isolation mixture of the following composition: 0.32 M sucrose, 1 mM EDTA, 10 mM Tris-HCl buffer, pH 7.5, using Ultra-Turrax T 10 homogenizer at a low speed, to obtain 30% *w*/*v* homogenate. Rat brain mitochondria were isolated as described in [42]. In each experiment we used brains from 5 animals.

### 4.3. Biotinylated Ubiquitin

Biotinylated ubiquitin was prepaed following the protocol developed for antibody biotinylation [43].

### 4.4. SDS-PAGE

SDS-PAGE was performed according to Laemmli in 12% gel [44].

### 4.5. Sample Preparation

Mitochondria (protein concentration, measured by the method of Bradford [45] was 2 mg/mL) were incubated with biotinylated ubiquitin (30 min, 37 °C) in 1 mL of incubation mixture containing 50 mM Tris-HCl, pH 7.5, 2 mM MgCl_2_, 3 mM dithiotreitol, 4 mM ATP, ATP-regenerating system (2 ME/mL creatine phosphokinase and 10 mM creatine phosphate), biotinylated ubiquitin (10 μM). The control mitochondria were incubated without ubiquitin. The reaction was terminated by cooling and subsequent centrifuging the incubation mixture (30 min, 16,000*g*).

### 4.6. Avidin Agarose Chromatography

Avidin agarose chromatography was performed to enrich the samples before the identification of ubiquitinated proteins. The mitochondrial pellets obtained at the previous stage (about 2 mg of protein) were resuspended in 100 μL of calcium/magnesium-free PBS (CMF-PBS; 2.68 mM KCl, 1.5 mM K_3_PO_4_ (monobasic), 136.9 mM NaCl, 8.1 mM Na_3_PO_4_ (dibasic heptahydrate)), pH 7.4, containing 3% Triton X-100, and after 45 min incubation at 4 °C were dissolved in the same buffer (without Triton X-100) containing bacitracin and aprotinin. The final concentration of Triton X-100 was 1%, bacitracin, 0.01% and aprotinin, 0.03%. Three hundred microliters of washed avidin-agarose slurry was then added to each sample and after 1 h incubation (25 °C) the slurry was washed 10 times with CMF-PBS containing 0.5 M NaCl and 2 times with CMF-PBS.

### 4.7. Proteomic Identification of Ubiquitinated Proteins

Avidin agarose absorbed proteins were modified by reduction with 0.02 M DTT in 6 M guanidine hydrochloride, pH 6.8, (1 h, 37 °C), and subsequent carbamidomethylation of SH groups with 0.055 M iodoacetamide (1 h, 37 °C). After the same extraction procedure [46] the protein sediment was dissolved in 50 mM ammonium hydrocarbonate and sonicated in the sonication bath for 15 min at 4 °C. After trypsinolysis (1 mg trypsin/100 mg of sample protein, 37 °C, 1 h; then 2 mg trypsin/100 mg of sample protein, 37 °C, overnight) formic acid was added to the preparations up to 0.5%. The preparations were evaporated using a vacuum concentrator 5301 (Eppendorf, AG, Germany), then dissolved in 0.1% formic acid, and analyzed by LC-MS/MS. Reverse-phase nano-LCMS/MS was performed using an Agilent 1100 nano-flow HPLC-Chip cube system coupled to Agilent 6340 Ion Trap (Agilent Technologies, Palo Alto, CA, USA). Tryptic peptides were separated on the HPLC Chip (40 nL trap column, 75 mm × 43 mm analytical column, 5 mm C-18SB-ZX, Agilent Technologies) using a linear gradient of 5%–80% ACN in 0.1% formic acid over 60 min at a flow rate of 300 nL/min and detected by an ion trap in 300–1800 *m*/*z* range following the supplier’s recommendations. Mass spectra were acquired in positive-ion mode with automated data-dependent MS/MS on the five most intense ions from precursor MS scans. Protein identification was performed using Spectrum Mill MS Proteomics Workbench Rev A.03.03.078 (Agilent Technologies). Protein identifications were obtained by comparison of experimental data to the Swiss-Prot rat and mouse subset database. The following search parameters were used: trypsin was used as the cutting enzyme, mass tolerance for the monoisotopic peptide window was set to 1.6 Da, the MS/MS tolerance window was set to 0.5 Da, and two missed cleavage were allowed. Cysteine carbamidomethylation was chosen as a fixed modification and oxidized methionine was chosen as a variable modifications. The criteria of positive identification were set as follows: 70% minimum scored peak intensity, d-forward reverse score 42; at least two peptides identifications with a confident score 7 and summarized protein score 14 [47].

Each protein listed in Tables 1–3 was identified at least in three independent experiments, each of which employed independent brain samples as well as their chromatographic and proteomic processing.

### 4.8. Identification of Ubiquitination Sites

Peptides with attached mono-ubiquitin and poly-ubiquitin moieties were searched against SwissProt Human proteins database. Branched and unbranched –GG signature peptide ions with the charge state of 2^+^, 3^+^ and 4^+^ generated by incomplete trypsin digestion were targeted for analysis by narrow (+/− 0.3 *m*/*z*) window extracted ion chromatography. Tandem MS/MS spectra of branched fragment ions shifted to the *m*/*z* value corresponding to the GG-moeity were isolated and analyzed for direct searching of ubiquitin-labeled peptides in samples. At least four branched fragment ions of the precursor with GG signature have to be founded for confident approval of ubiquitin-labeled peptides.

### 4.9. Prediction of a Mitochondrial Interactome/Protein Interaction Network

Prediction of interactions of proteins identified during the proteomic analysis was performed using an open access STRING version 9.0 software platform [48]. A list of identified proteins Uniprot/SwissProt IDs was used as input data and applied for interactive network processing. The confidence score at the level of 0.7 was chosen to view the best fitted proteins with approximate probability that a predicted link exists between selected proteins in the same metabolic map in the KEGG database. KMEANS clustering algorithm was selected for clustering the obtained proteins in the resultant network. The number of clusters was chosen as 5 and at least the confidence score of 0.9 was selected for each analyzing node in the resultant protein connectivity map.

## 5. Conclusions

Thus, results of the present study demonstrate that the use of biotinylated ubiquitin may be considered as the method of choice for *in vitro* evaluation of endogenous ubiquitin-conjugating machinery in particular subcellular organelles and changes in ubiquitin/organelle associated interactomes. We believe that our approach is a good supplement to traditionally used methods for detection of direct ubiquitination targets and evaluation of ubiquitin conjugated activity known in the literature [49–52].

This may be useful for evaluation of changes in mitochondrial interactomes induced by protein ubiquitination under normal and various brain pathologies, which still require better characterization.

## Figures and Tables

**Table 1:** Proteomic identification of rat brain ubiquitin binding proteins: M, EM, PM designate mitochondrial, extramitochondrial and plasma membrane localization, respectively; “?” precise localization remains unknown at the moment. Here and in subsequent Tables each protein was identified at least in three independent experiments.

No.	Protein name	Database Accession Number	Sequence coverage (%)	Search score	Localization
Proteins/enzymes involved in energy generation and carbohydrate metabolism (*n* = 13)					

1	Pyruvate carboxylase, mitochondrial precursor	P52873	25	285.19	M
2	ATP synthase subunit beta, mitochondrial precursor	P10719	47	231.07	M
3	ATP synthase subunit alpha, mitochondrial precursor	P15999	30	163.32	M
4	Sodium/potassium-transporting ATP-ase subunit alpha-3	P06687	13	130.82	PM
5	Glyceraldehyde-3-phosphate dehydrogenase	P04797	14	48.89	EM
6	ADP/ATP translocase 2	Q09073	11	42.59	M
7	Creatine kinase M-type	P00554	12	56.1	M
8	Sodium/potassium-transporting ATP-ase subunit alpha-2 precursor	P06686	5	37.89	PM
9	ATP synthase subunit O, mitochondrial precursor	Q06647	11	34.75	M
10	Sodium/potassium-transporting ATP-ase subunit alpha-1, precursor	P06685	2	21,46	PM
11	ATP synthase subunit d, mitochondrial	P31399	10	16.97	M
12	Pyruvate dehydrogenase E1 component subunit beta, mitochondrial precursor	P49432	4	15.32	M
13	Dihydrolipoyllysine-residue acetyltransferase component of pyruvate dehydrogenase complex, mitochondrial precursor	P08461	2	14.02	M

Proteins involved in cytoskeleton formation and exocytosis (*n* = 24)					

1	Keratin type I cytoskeletal 10	Q6IFW6	19	176.37	EM
2	Keratin type II cytoskeletal 5	Q6P6Q2	14	165.65	EM
3	Keratin type II cytoskeletal 1	Q6IMF3	9	124.82	EM
4	Keratin type I cytoskeletal 42	Q6IFU7	16	118.43	EM
5	Keratin type II cytoskeletal 6A	Q4FZU2	13	115.79	EM
6	Tubulin alpha-1B chain	Q6P9V9	27	111.39	EM
7	Keratin type I cytoskeletal 14	Q6IFV1	9	80.83	EM
8	Neurofilament light polypeptide	P19527	13	71.54	EM
9	Tubulin beta-2B chain	Q3KRE8	12	62,92	EM
10	Tubulin beta-2A chain	Q85108	12	62,92	EM
11	Glial fibrillary acidic protein	P47819	14	57.15	EM
12	Tubulin beta-5 chain	P69897	12	54.58	EM
13	Tubulin beta-3 chain	Q4QRB4	10	48.33	EM
14	Myosin-4	Q29RW1	2	45.07	EM
15	Alpha-internexin	P23565	11	43.84	EM
16	Actin, alpha skeletal muscle	P68136	11	43.17	EM
17	Tubulin alpha-1B chain	Q6P9V9	9	39.35	EM
18	Tubulin alpha-1A chain	P68370	9	39.35	EM
19	Myosin-6	P02563	2	37.23	EM
20	Neurofilament medium polypeptide	P12839	5	35.80	EM
21	Myosin-3	P12847	1	34.23	EM
22	Syntaxin-1B	P61265	10	26.19	EM
23	Synaptotagmin-1	P21707	3	18.57	EM
24	Spectrin alpha chain, brain	P16086	1	17.02	EM

Protective proteins (*n* = 1)					

1	60 kDa heat shock protein, mitochondrial precursor	P63039	9	29.87	M

Enzymes involved in signal transduction and regulation of enzyme activity (*n* = 3)					

1	Calcium/calmodulin-dependent protein kinase	P11275	7	39.65	EM
2	2′,3′-cyclic-nucleotide 3′-phosphodiesterase	P13233	8	46.16	EM
3	Pro-associated protein kinase 2	Q62868	1	19.53	EM

Enzymes involved in fatty acid metabolism (*n* = 2)					

1	Propionyl-CoA carboxylase beta chain, mitochondrial precursor	P07633	13	67.94	M
2	Propionyl-CoA carboxylase alpha chain, mitochondrial precursor	P14882	5	37.33	M

Transporters (*n* = 4)					

1	Excitatory amino acid transporter 1	P24942	6	34.57	PM
2	Excitatory amino acid transporter 2	P31596	2	26.35	PM
3	Glial high affinity glutamate transporter isoform a	G3V6R2	2	25.7	PM
4	sodium-dependent high affinity glutamate transporter GLT-1A	G3V6R0	2	26.0	PM

Ubiquitin and related proteins (*n* = 3)					

	polyubiquitin	P0CG51	51	54.99	EM/M ?
	ubiquitin C	Q63429	4	44.01	EM/M ?
	Ubc protein	Q5FWT0	10	44.01	EM/M ?

**Table 2:** Proteomic identification of proteins from control rat brain mitochondria incubated without biotinylated ubiquitin.

No	Protein Name	Database Accession Number	Sequence Coverage (%)	Search Score
1	rCG50775	EDL86881	11	102.68
2	type I keratin KA17	Q6IFU8	14	88.22
3	cytokeratin-8	Q80WH8	9	86.42
4	keratin complex 2, basic, gene 8	Q10758	9	86.42
5	hypothetical protein LOC683313	Q63282	11	85.15
6	rCG50690	EDL86882	27	76.25
7	Keratin, type II cytoskeletal 75	Q6IG05	11	71.38
8	keratin complex 1, acidic, gene 14	NP_001008751	8	63.05
9	type I keratin KA22	Q6IFU7	9	62.98
10	similar to keratin complex 2, basic, gene 6a isoform 1	NP_001094477	7	61.64
11	rCG50520	EDL86873	4	60.01
12	rCG23467, isoform CRA a	G3V8B0	2	59.00
13	rCG23609, isoform CRA b	EDM14209	2	59.00
14	type II keratin Kb15	Q6IFZ4	7	56.92
15	type I keratin KA15	Q6IFV3	8	56.63
16	type II keratin Kb39	Q6IG01	4	56.21
17	rCG34382, isoform CRA_a	G3V6D8	2	56.00
18	type I keratin KA16	Q6IFU9	9	55.18
19	rCG33578	EDM06024	12	52.72
20	type II keratin Kb36	Q6IG03	6	46.15
21	type II keratin Kb1	Q6IMF3	7	45.54
22	type I keratin KA11	Q6IFV0	5	45.00
23	Krt1–19 protein	Q63279	5	41.32
24	keratin complex 1, acidic, gene 19, isoform CRA b	EDM06018	8	41.32
25	rCG34382, isoform CRA b	EDM04783	2	40.63
26	rCG33626	EDM04786	2	37.50
27	myelin basic protein, isoform CRA a	Q63327	10	34.60
28	rCG23467, isoform CRA b	EDM14212	2	29.34
29	PREDICTED: similar to type II keratin Kb36	NP_001008808	6	28.42
30	keratin complex 1, acidic, gene 19, isoform CRA c	EDM06019	6	27.10
31	myelin basic protein isoform 1	P02688-1	10	27.03
32	myelin basic protein	Q80Z98	10	27.03
33	myelin basic protein isoform 2	P02688-2	12	27.03
34	myelin basic protein isoform 4	P02688-4	13	27.03
35	rCG34505	EDM05987	2	25.35
36	myelin basic protein, isoform CRA d	Q5XFW	7	23.60
37	myelin basic protein, isoform CRA c	G5E945	8	23.60
38	keratin 13	Q6IFV4	3	23.44
39	rCG33575	EDM04790	2	22.89
40	proteolipid protein	P63081	7	20.85
41	type II keratin Kb2	Q6IG02	2	20.30
42	Tubulin, alpha 1A	P68370	5	19.83
43	tubulin, alpha 1C	Q6AYZ1	5	19.83
44	rCG50513, isoform CRA b	EDL87016	7	19.83
45	rCG50513, isoform CRA a	EDL87015	7	19.83
46	keratin complex 1, acidic, gene 12	Q5BJY9	1	17.88
47	rCG34869	EDM05985	3	17.88
48	keratin 19	Q63279	3	17.88
49	type II keratin Kb9	Q6IFZ5	3	17.11
50	rCG62531, isoform CRA d	EDM00590	9	16.42
51	myelin basic protein isoform 3	P02688-3	6	16.03
52	myelin basic protein isoform 5	P02688-5	7	16.03
53	rCG44184, isoform CRA a	EDL98317	4	15.98
54	rCG29914, isoform CRA b	EDM01924	5	15.23
55	Tpi 1 protein	P48500	6	15.23
56	rCG29914, isoform CRA a	EDM01923	6	15.23
57	rCG47746, isoform CRA b	EDM13157	13	14.99
58	keratin 20	P25030	2	14.22
59	rCG33887, isoform CRA a	EDM06013	14	14.22

**Table 3:** Proteomic identification of rat brain proteins containing ubiquitin signatures.

No	Protein Name	Database Accession Number	Acronym name	No. of peptides	Sequence Coverage (%)	Search score	Sequence/position of a.a. residue/*
1	Pyruvate carboxylase, mitochondrial precursor	P52873	Pc	18	25	285.19	SGEGMGIRLDNASAFQGAVISPHYDSLLVKVIAHG KDHPTAAT***K***MSR/**K442**/EV***K***KAYVEANQMLGDLIKVTPSSKIVGDLAQFMV QNGLSR/**K891**/SSTAPVASPNVRRLEY***K***PIKKVMVANR/**K35**/
2	ATP synthase subunit alpha, mitochondrial precursor	P15999	Atp5a1	11	30	163.32	ELIIGDRQTG***K***TSIAIDTIINQK/**K218** VGSAAQTRAmKQVAGTM***K***LELAQYREVAAFAQF GSDLDAATQQLLSR/**K434**/VVDALGNAIDG***K***GPVGSKIR/**K161**/AM***K***QVAGTMKLELAQYREVAAFAQFGSDLDAAT QQLLSR/**K427**/
3	Sodium/potassiumtransporting ATP-ase subunit alpha-3	P06687	Atp1a3	9	13	130.82	EKLENMKKEMEMNDHQLSVSELEQ***K***YQTSATK/**K 63**/RDTAGDASESALL***K***CIELSCGSVRKMR/**K456**/GFAFDCDDVNFTTDNLCFVGLMSMIDPPRAAVPDA VG***K***CR/**K595**/
4	Keratin type I cytoskeletal 10	Q6IFW6	Krt10	14	19	176.37	NELTEM***K***RTLQTLEIELQSLLAMK/**K298**/TIEDLKS***K***ILAATVDNANVLLQIDNAR/**K182**/
5	Keratin type II cytoskeletal 5	Q6P6Q2	Krt5	20	14	165.65	IKTQEREQI***K***TLNNKFASFIDK/**K172**/EYQELMNT***K***LALDVEIATYR/**K456**/NKYEDEIN***K***RTEmENEFVLIKK/**K185**/ALNNKFASFIDKVRFLEQQNQVLET***K***WELLQQLDQ NNSR/**K165**/
6	Keratin type II cytoskeletal 1	Q6IMF3	Krt1	9	9	124.82	LALDMEIATYR***K***LLEGEEIRMSGECTPNVSVSVSTS HTSMSGTSSR/**K483**/MLREHQELMSM***K***LALDIEIATYR/**K423**/TTAENEFVVL***K***KDVDAAYMSKVELQAKVDALDGE IK/**K139**/
7	Myosin-4	Q29RW1	Myh1	4	2	45.07	NTQGML***K***DTQLHLDDALRGQDDLKEQLAMVERR/**K1655**/I***K***KKMEGDLNEMEIQLNHANRQAAEAIR/**K1619**/SGLYKLTGAVMHYGNMKF***K***QK/**K366**/KLEGDLKLAQESTMDIENDKQQLDE***K***LK/**K1083**/AFMGVKNWPWMKLYFKI***K***PLLK/**K838**/FQLEA***K***IKEVTERAEDEEEINAELTAK/**K919**/KMEGDLNEMEIQLSHANRMAAEAQKQV***K***EAR/**K1 644**/GKAEAHFSLVHYAGTVDYNIIGWLD***K***NK/**K599**/
8	Actin, alpha skeletal muscle	P68136	Acta2	3	11	43.17	***K***DLYANNVMSGGTTMYPGIADRMQKEITALAPST MK/**K293**/AVFPSIVGRPRHQGVMVGMGQ***K***DSYVGDEAQSKR/**K50**/
9	Myosin-6	P02563	Myh6	3	2	37.23	QREEQAEPDGTEDAD***K***SAYLMGLNSADLLK/**K383**/
10	Spectrin alpha chain, brain	P16086	Spna2	2	1	17.02	MQHNLEQQIQARNTTGVTEEALKEFSMMF***K***HFDK/**K2333**/SCKKFMLFREANELQQWINE***K***EAALTSEEVGADLE QVEVLQK/**K1110**/DLNSQADSLMTSSAFDTSQVKE***K***R/**K1738**/
11	2′,3′-cyclic-nucleotide 3′-phosphodiesterase	P13233	Cpn	3	8	46.16	G***K***LYSLGKGRWMLSLAKK/**K370**/
12	Excitatory amino acid transporter 2	P31596	Slc1a2	2	2	26.35	MASTEGANNMP***K***QVEVRMHDSHLSSEEPKHRNLGMR/**K12**/

* Positions of identified ubiquitinated residues are shown in bold italics and underlined.

Abbreviations used for Figure 2. Atp5f1 = P19511, Atp5h = P31399, Atp5c1 = P35435, Atp5e = P29418, Atp5o = Q06647, Atp5a1 = P15999, Atp5b = P10719, Atp5d = P35434, ATP6 = P05504, Krt10 = Q6IFW6, Krt1 = Q6IMF3, Krt5 = Q6P6Q2, Myh1 = Q29RW1, Acta2 = P68136, Myh6 = P02563, Spna2 = P16086, Cpn = P13233, Slc1a2 = P31596, Atp1a3 = P06687, Pc = P52873, Krt42 = Q6IFU7, Pdhx = Q5BJX2, 686201 =Q4FZZ4, Pdhb = P49432, Dlst = Q01205, Dbt = B2GV15, Pdha1 = P26284, Dlat = P08461, Hspd1 = P63039, Hspe1 = P26772, Slc25a5 = Q09073, Pcca = P14882, Pccb = P07633, Atp1a2 = P06686, Atp1a1 = P06685, Nefm = P12839, Nefl = P19527, Camk2a = P11275, Ina = P23565, Gfap = P47819, Slc1a3 = P24942, ENSRNOG00000018630 = Q0QEU1, Krt14 = Q6IFV1, Tubb3 = Q6P9T8, Tuba4a = Q6P9V9, Tubb5 = Q3KRE8, Vamp2 = P63045, Snap23 = O70377, Snap25 = P60881, Stx1b = P61265, Syt1 = P21707.
